# Acute Neurological Manifestations of COVID-19 Patients From Three Tertiary Care Hospitals in Qatar

**DOI:** 10.7759/cureus.23150

**Published:** 2022-03-14

**Authors:** Liaquat Ali, Ahmad Muhammad, Adnan Khan, Imran Mohammed, Imran Janjua, Yasin Zada, Muhammad Sharif, Muhammad Naeem, Ambreen Iqrar, Khawaja Hassan Haroon

**Affiliations:** 1 Neurology, Neurosciences Institute, Hamad General Hospital, Doha, QAT; 2 Neurology, Weill Cornell Medicine Qatar, Doha, QAT; 3 Ophthalmology, Weill Cornell Medicine Qatar, Doha, QAT; 4 Internal Medicine, Hamad General Hospital, Doha, QAT; 5 Neurology, Hamad General Hospital, Doha, QAT; 6 Neurosciences, Hamad General Hospital, Doha, QAT

**Keywords:** intensive care unit- acquired weakness (icu-aw), creatine kinase (ck), acute disseminated encephalomyelitis (adem), acute respiratory distress syndrome (ards), severe acute respiratory syndrome coronavirus 2 (sars-cov-2), coronavirus disease 2019 (covid-19)

## Abstract

Introduction

Worldwide, there are more than 424 million confirmed cases of COVID-19. Most of the hospitalized critical COVID-19 patients manifested neurological signs and symptoms and higher mortality. The majority of COVID-19 fatalities occurred mostly in patients with advanced age and underlying medical comorbidities. This is the first local retrospective study in Qatar, which reported neurologic manifestations (48.5%) of hospitalized COVID-19 patients. The primary objective of this study is to evaluate acute neurological manifestations in COVID-19 hospitalized patients in the country.

Methods

This is a retrospective, observational study of 413 hospitalized COVID-19 patients. They were admitted to three different COVID-19 designated hospitals (Hazm Mebaireek, Ras Laffan, and Cuban tertiary care Hospitals) under the Hamad Medical Corporation, Qatar from 1st January 2020, to 31 January 2021. We evaluated electronic medical records of these patients and data were collected while their neurological manifestations were confirmed by two trained neurologists. These neurologic manifestations were categorized into three major groups: central nervous system (CNS), peripheral nervous system (PNS), and neuromuscular system.

Results

Of 413 patients, 94% (389) were male and 6% (24) were female; the mean age was 52 years. Among all different nationalities of COVID-19 patients, 20.3% (84) were Indian, 12.5% (52) were Bangladeshi, 10.1% (42) were Qatari and 9.2% (38) were Nepali. The most common symptoms at the onset of COVID-19 illness were as follows: 77.5% (321) had a fever, 67.4% (279) experienced cough, 58.7% (243) experienced shortness of breath and 26.1% (108) developed a sore throat. Overall 48.5% (201) patients developed different neurologic manifestations. The most common neurologic symptoms were myalgia (28%; 116), headache (10.4%; 43), dizziness (5.8%; 24) and hemiparesis due to strokes (5.3%; 22). In this study, the most common risk factors were hypertension (47.6%), diabetes (46.9%), obesity (21%), chronic kidney disease (10%), ischemic heart disease (9.7%), and smoking (6.8%). About 45.2% (187) patients were admitted to MICU and 8.5% (35) died due to COVID-19 complications. Significant other extrapulmonary multiorgan system involvement were skeletal muscle injury (39.4%), kidney injury (36.7%), liver injury (27.5%), myocardial injury (23.9%), rhabdomyolysis (15.7%) heart failure (11.4%) and acute pancreatitis (11.1%).

Discussion

The most common neurologic signs and symptoms were myalgia, headache, dizziness, and strokes, mainly due to large vessel thrombosis, lacunar, and posterior circulation strokes.

Conclusions

Patients with COVID-19 are at high risk of developing neurological manifestations. The most common COVID-19-related acute neurological manifestations were myalgia, headache, dizziness, and acute ischemic stroke. Prompt recognition, early diagnosis, and appropriate management of these manifestations could potentially lead to better patient outcomes in COVID-19 patients.

## Introduction

In late December 2019, several patients with viral pneumonia were diagnosed in Wuhan, Hubei Province of China. This virus rapidly involved several provinces of China and many countries in the world. WHO declared a pandemic in March 2020 due to this novel virus called severe acute respiratory syndrome coronavirus 2 (SARS-CoV-2) [1].

The spectrum of symptomatic infection ranges from mild to critical. The global case fatality rate of COVID-19 is 2-3% and most of the fatalities occurred in patients with advanced age or underlying medical comorbidities. However, there is an estimated asymptomatic infection rate as high as 30 to 40% in the community [2]. 

Several studies have described typical COVID-19 clinical manifestations such as fever, cough, dyspnea, fatigue, and diarrhoea. There have been few studies describing neurological manifestations in COVID-19 patients. However, to our knowledge, this is the first large case series in the six Gulf Cooperation Council (GCC) countries (Qatar, KSA, UAE, Kuwait, Oman, and Bahrain) to report the characteristic neurological manifestations of COVID-19 infection in 48.5% (201) of 413 patients with a laboratory-confirmed diagnosis of COVID-19, treated at three tertiary care hospitals in Qatar.

## Materials and methods

This is a retrospective, observational cross-sectional study, conducted at three COVID-19 designated government hospitals (Hazm Mebaireek, Ras Laffan, and Cuban) of the Hamad Medical Corporation (HMC), Doha, Qatar. The data of 413 consecutive COVID-19 patients, confirmed by reverse transcription-polymerase chain reaction (RT-PCR), admitted during the first wave from 1st January 2020, to 31 January 2021 were retrospectively analyzed.

Electronic medical records including laboratory and radiologic findings of all patients with confirmed SARS-CoV-2 infection were evaluated and data were collected on age, gender, nationality, symptoms (fever, cough, SOB, myalgia, headache), comorbidities (hypertension, diabetes, obesity, ischemic heart disease, chronic kidney disease, and smoking). We defined the degree of severity of COVID-19 (mild vs severe) at the time of admission using the American Thoracic Society guidelines for community-acquired pneumonia.

Neurologic manifestations were categorized into three major groups: central nervous system (CNS) manifestations (included dizziness, headache, impaired consciousness/encephalopathy, acute cerebrovascular disease, and seizure), peripheral nervous system (PNS) manifestations (included anosmia, dysgeusia, Guillain-Barre syndrome (GBS) and nerve injury), and neuromuscular system manifestations (including skeletal muscular injury). All neurological manifestations were checked by two trained neurologists.

Routine laboratory testing (complete blood count (CBC), prothrombin time (PT), activated partial thromboplastin time (APTT), urea, creatinine, electrolytes, liver function test (LFT), C-reactive protein (CRP), ferritin, lactate dehydrogenase (LDH), D-dimer, interleukin-6 (IL-6), lactic acid, procalcitonin, creatine kinase (CK), myoglobin, troponin-T), radiological (chest x-ray, CT thorax, CT and CTA head and neck, MRIs and MRAs of the head and neck), cardiac (ECGs, echocardiograms) and others investigations (EMG/NCS studies and lumbar puncture) were performed according to standard clinical practice and CDC Qatar guidelines.

The study was performed according to the principles of the Declaration of Helsinki. This study was approved and written informed consent was waived by the Medical Research Center of HMC, Doha, Qatar, in August 2020 due to the COVID-19 pandemic and urgent need to collect and analyze new data to identify gaps in practice and improve patient outcomes.

The primary objective of this study was to determine the prevalence of acute neurological manifestations and the secondary objective was to determine other extrapulmonary multiorgan system involvement in COVID-19 patients from three tertiary care hospitals of Qatar.

## Results

This study comprised 413 hospitalized COVID-19 patients confirmed by RT-PCR for SARS-CoV-2. Their mean age was 52 years (standard deviation 14 and ranging from 22 to 86 years), and 94% were men (389) and 6% were female (24). Their demographic, risk factors for COVID-19, and clinical characteristics are as shown in Table 1.

**Table 1:** Demographic, risk factors for COVID-19 and clinical characteristics

Age(years) (n=413)	Frequency(n)	Percentage (%)
<55 year	234	56.7
>55 year	179	43.3
Gender		
Male	389	94
Female	24	6
Nationality		
Indian	84	20.3
Bangladesh	52	12.5
Qatari	42	10.1
Nepalese	38	9.2
Filipino	36	8.7
Pakistani	36	8.7
Others (22 nations)	125	30.5
Risk factors		
Hypertension	197	47.6
Diabetes	196	46.9
Obesity	88	21
Newly diagnosed DM	42	10.1
Chronic Kidney disease	43	10.4
Ischemic heart disease	40	9.7
Smoker	28	6.8
Asthma	15	3.6
Malignancy	12	2.9
Clinical manifestations		
Non-Neurological Symptoms		
Fever	321	77.5
Cough	279	67.4
SOB	243	58.7
Sore throat	108	26.1
Nausea	96	23.2
Vomiting	48	11.6
Abdominal discomfort	47	11.3
Chest pain	41	9.9
Dysuria	6	1.4
Neurological Manifestations	201	48.5
CNS		
Headache	43	10.4
Dizziness	24	5.8
Stroke	22	5.3
CVST	03	0.7
Seizure	02	0.5
PNS		
ICU acquired weakness	13	3.1
Anosmia	06	2.9
Dysgeusia	12	1.4
Neuromuscular system		
Skeletal muscle injury	163	39.4
Myalgia	116	28
Rhabdomyolysis	65	15.7

The most common nationalities with COVID-19 were 84 (20.3%) Indian, 52 (12.5%) Bangladeshi, 42 (10.1%) Qatari and 38 (9.2%) Nepali. The most common symptoms at the onset of COVID-19 illness were fever (321; 77.5%), cough (279; 67.4%), shortness of breath (243; 58.7%) and sore throat (108; 26.1%). The other important symptoms were nausea (96; 23.2%), vomiting (48; 11.6%), abdominal discomfort (47; 11.3%) and chest pain (41; 9.9%). The most common underlying risk factors were hypertension (197; 47.6%), diabetes mellitus (194; 46.9%), obesity (88; 21%), chronic kidney disease (43; 10%), ischemic heart disease (40; 9.7%) and smoking (28; 6.8%) as shown in Table 1.

Of 413 COVID-19 patients, overall 48.5% (201) patients had neurological manifestations (Table 1). Neurological manifestations were divided into three major categories: central nervous system (CNS), peripheral nervous system (PNS), and neuromuscular system; 39.4% were neuromuscular system manifestations while CNS and PNS manifestations were 26.7% and 4.3% respectively, as shown in Table 1.

For patients with CNS manifestations, the most common presentations were headache (43; 10.4%), dizziness (24; 5.8%), and strokes (22; 5.3%). For patients with PNS manifestations, the most common manifestations recorded were intensive care unit-acquired weakness (13; 3.1%), anosmia (12; 2.9%), and dysgeusia (6; 1.4%). In relation to the neuromuscular system, the most common manifestations were skeletal muscle injury with evidence of creatine kinase >300U/L (163; 39.4%), myalgia (116; 28%) and rhabdomyolysis (65; 15.7%) with evidence of creatine kinase >1000 U/L as shown in Table 1.

**Figure 1:**
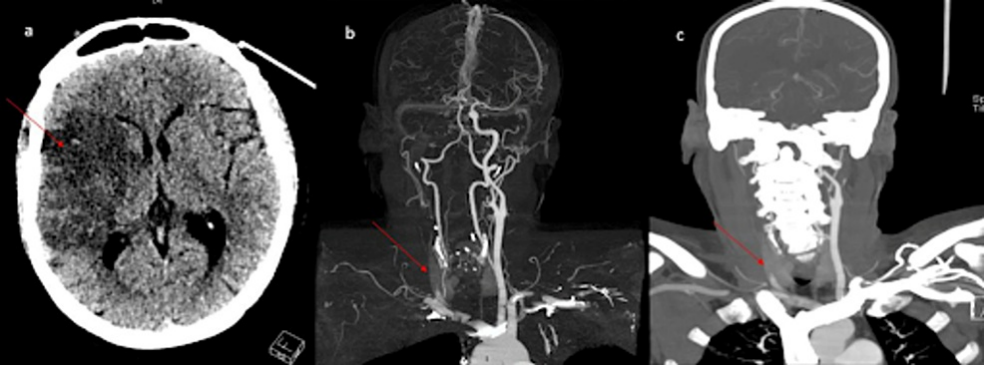
Non-contrast CT scan head and cerebral angiogram showing right side large vessel middle cerebral artery ischemic stroke of a COVID-19 patient. Non-contrast CT scan of the head shows a large vessel ischemic infarct in the right middle cerebral artery territory (a). CT cerebral angiogram and MIP images (as shown in b, c) show complete occlusion of right common carotid artery starting from its proximal end with non-visualization of external common carotid, internal carotid, and middle cerebral artery (b, c).

**Figure 2:**
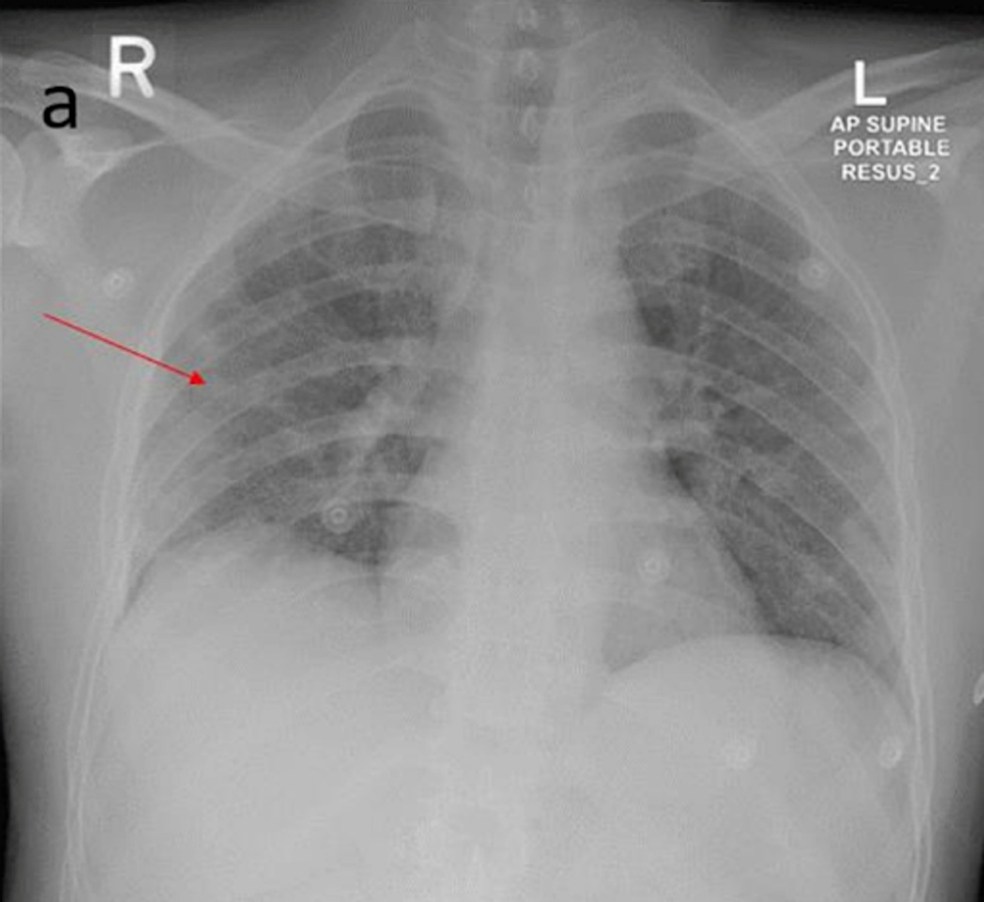
Chest X-ray (AP) of COVID-19 patient Chest X-ray shows patchy peripheral ground-glass opacities in the bilateral lung fields.

Additionally, 36 (8.7%) patients had CNS involvement in the form of different types of strokes such as large vessel thrombosis, lacunar, posterior circulation, cardioembolic ischemic strokes, ICH as well as leukoencephalopathy with or without microhemorrhages and hypoxic-ischemic cerebral injury (as shown in Table 2) as evident on brain neuroimaging. Moreover, 22 (5.3%) patients either had ischemic (19; 4.6%) or hemorrhagic (3; 0.7%) stroke. The most common cause of stroke was large artery thrombosis (8; 1.9%) as shown in CT head and CT cerebral angiogram images of a COVID-19 patient as shown in Figures 1A-IC; the chest X-ray (Figure 2) shows bilateral patchy peripheral ground-glass opacities in lung fields

**Table 2:** Different types of strokes in COVID-19 patients Around 8.7% patients had different types of strokes, including large vessel stroke, lacunar, posterior circulation, cardio-embolic ischemic strokes, ICH as well as leukoencephalopathy with or without microhemorrhages, and hypoxic ischemic cerebral injury.

Types of Strokes	Frequency(n)	Percentage (%)
Large artery Thrombosis Stroke	8	1.9
leukoencephalopathy with or without microhemorrhages	7	1.7
Lacunar Stroke	5	1.2
Hypoxic ischemic cerebral injury	4	1.0
Posterior Circulation Ischemic Stroke	4	1.0
Cerebral venous sinus thrombosis	3	0.7
Intracerebral Hemorrhage (ICH)	3	0.7
Cardiogenic Embolic Stroke	2	0.5
Total	36	8.7

Of a total of 413 patients, 187 (45.2%) patients were admitted to the medical intensive care unit (MICU) with critical COVID-19 illness and 120 (29%) patients needed mechanical ventilation while 67 (16.2%) required non-invasive ventilation. In MICU-admitted patients, the mortality rate was 8.5% (35) due to COVID-19 related complications. Of 187 MICU patients, 13 (7%) developed intensive care unit-acquired weakness or critical illness neuromyopathy.

Other non-neurological extra-pulmonary multiorgan system involvement in COVID-19 patients were kidney injury (152; 36.7%), hepatic injury (114; 27.5%), myocardial injury (99; 23.9%), heart failure (47; 11.4%), and pancreatitis (46; 11.1%) while 24 (5.8%) had uncontrolled blood sugar as shown in Table 3. 

**Table 3:** Extrapulmonary multiorgan system involvement in 413 COVID-19 patients. Conditions with extrapulmonary multiorgan system involvement in COVID-19 patients included kidney injury, hepatic injury, myocardial injury, rhabdomyolysis, heart failure and pancreatitis in descending order.

Multiorgan Systems	Frequency(n)	Percentage (%)
Acute kidney injury (AKI) (urea>8mmol/L, creatinine>106ml/min)	152	36.7
Acute liver injury (ALT or AST >200 U/L)	114	27.5
Acute myocardial injury (troponin-T hs; male>30, female>20 ng/l)	99	23.9
Rhabdomyolysis (serum CK>1000 U/L)	65	15.7
AHF (Pro-BNP >1800 pg/ml)	47	11.4
Acute pancreatitis (serum lipase >180 u/l)	46	11.1
Uncontrolled diabetes mellitus (HbA1c>12)	24	5.8

Serum ferritin, CRP, and IL-6 levels were high in 295 (71%), 253 (61%), and 153 (37%) patients respectively, indicative of severe COVID-19 illness. Around 223 (53.9%) had high D-dimers (>1000 ng/ml) indicative of COVID-19 related coagulopathy as shown in Table 4.

**Table 4:** Laboratory findings in COVID-19 patients. Laboratory findings showed high serum ferritin, CRP and IL-6 levels and D-dimer COVID-19 patients respectively, indicative of severe COVID-19 illness.

Lab: Findings	Frequency (n)	Percentage (%)
Serum Ferritin>500mcg/L	295	71
LDH >245 units/L	274	66.2
C-reactive protein levels	253	61
D-Dimer >1000 ng/ml	223	53.9
Serum CK>300u/L	163	39.4
IL6>70 pg/ml	153	37
Serum myoglobin >72 ng/dl	148	35.7
Procalcitonin > 2ng/ml	90	22.2
Serum CK>1000 U/L (Rhabdomyolysis)	65	15.7
Lactic acid > 4mml/l	26	6.3

## Discussion

In late December 2019, several patients with viral pneumonia were diagnosed in Wuhan, Hubei Province of China. This viral infection was epidemiologically associated with a seafood market where a number of non-aquatic animals such as birds and rabbits were also on sale before the outbreak [1, 3]. This rapidly involved several provinces of China and many countries in the world and hence WHO declared a pandemic in March 2020 due to a novel single-stranded RNA coronavirus, named SARS-CoV-2 [1].

As of February 2022, there are more than 424 million confirmed COVID-19 cases, including more than five million deaths worldwide [1]. Acute respiratory distress syndrome is a major complication in patients with severe COVID-19 infection [3]. COVID-19 has a 2-3% case-fatality rate globally [2] and most of the fatalities occurred in patients with advanced age or underlying medical comorbidities [4-6]. COVID-19 case-fatality rate in the country of Qatar is quite low at 0.255% as compared to the rest of the world. Several variants of SARS-CoV-2 have emerged notably because of the potential for increased transmissibility [7] and hence more complications. There have been few studies describing neurological manifestations in COVID-19 patients.

This is the first detailed retrospective, observational study of acute neurological manifestations in hospitalized COVID-19 patients in Qatar, and to our knowledge, this is the first study in the GCC countries to describe these findings in COVID-19 patients. Our population is relatively younger, and the majority were male (94%), mostly expatriates largely from underdeveloped countries. Around 28 nationalities were included in our study as people from over a hundred different nationalities are living in Qatar.

The Centers for Disease Control and Prevention (CDC), United States, reported cough, fever, and shortness of breath in 50%, 43%, and 29% in mild to severe COVID-19 patients respectively [8]. In a study of hospitalized COVID-19 patients, the most common symptoms at the onset of illness were fever (61.7%), cough (50.0%), and anorexia (31.8%) [9], while our study revealed the most common symptoms at onset were fever (77.5%), cough (67.4%) and shortness of breath (58.7%).

Neurological manifestations were observed in 36.4% of hospitalized patients which were more common in patients with severe infection and had higher mortality rates [9]. In our study, there were overall 48.5% (201/413) of patients with various neurologic manifestations including CNS, PNS and neuromuscular systems involvement. The most common neurological manifestations of the neuromuscular system were skeletal muscle injury, myalgia and rhabdomyolysis, while in CNS the most common manifestations were headaches, dizziness, strokes, CVST and seizures and in PNS the manifestations were ICU acquired weakness (ICU-AW), anosmia and dysgeusia in order of descending frequency.

Neurologic complications in COVID-19 patients may be due to multifactorial mechanisms that may arise from direct as well systemic responses to the virus [10]. In addition to traditional vascular mechanisms in COVID-19 patients, dysfunction of the renin-angiotensin system (RAS) due to virus binding to angiotensin-converting enzyme -2 (ACE2), hypercoagulability, severe inflammation, and cardiac dysfunction may lead to secondary cardiovascular and cerebrovascular effects [11-13]. Critically ill COVID-19 patients may develop hyperinflammation, endothelialitis, cytokine storm, thrombophilia, and complement activation [14, 15] leading to thromboembolic disease. SARS-CoV-2 may invade the brain through the olfactory epithelium and neural-mucosal interface. In addition to potential direct SARS-CoV-2 viral myositis, cytokines storm could also lead to pathological changes in skeletal muscle tissue.

Toxic-metabolic encephalopathy and strokes are common acute neurological manifestations in critically ill COVID-19 patients with other rare case reports of Guillain-Barre syndrome (GBS), acute disseminated encephalomyelitis (ADEM), meningoencephalitis, generalized myoclonus, and posterior reversible encephalopathy syndrome (PRES). In a study of 509 hospitalized COVID-19 patients, 31.8% had encephalopathy [16]. A spectrum of neuroimaging abnormalities has been described in patients with COVID-19, the most common being acute ischemic stroke, cortical FLAIR signal abnormality, cerebral microbleeds, leptomeningeal enhancement, cytotoxic lesions in the splenium of the corpus callosum, and other manifestations of encephalitis [17].

We had 10.4% of patients with headaches in our study which is comparable to another study that reported 13% [9]. US CDC data recorded headache in 34% of patients [8] while they had only 14% hospitalized patients. The incidence of ischemic and hemorrhagic strokes in COVID-19 hospitalized patients have ranged from 0.4 to 2.8% and 0.2 to 0.9% respectively [9, 18, 19] while our study showed 4.6% ischemic and 0.7% hemorrhagic strokes. In our study, the rate of ischemic stroke is high which is likely due to an overall higher number of patients with known underlying vascular risk factors and severe COVID-19 illness leading to thromboembolic complications [13]. 

Around 3.1% (13) patients developed intensive care unit-acquired weakness (ICUAW) which comprises critical illness myopathy (CIM), critical illness polyneuropathy (CIP), and a combination of both (CIMP). Three patients had CIM and seven patients had a combination of both critical illness myopathy and critical illness polyneuropathy (CIMP) clinically supported by electrophysiological studies, while three patients were diagnosed clinically as ICUAW after excluding brain and spinal cord pathology as NCS/EMG studies were not possible. None of the patients' samples were sent for biopsy. Among MICU admitted patients, ICUAW developed in 7% of our patients. We do not know the exact incidence of ICUAW in critically ill COVID-19 patients, but one study has shown that 9.9% of the patients developed ICUAW [20].

Anosmia and dysgeusia have been reported in more than 80% of the patients as early symptoms in mild to moderate COVID-19 illness [21, 22]. Loss of smell or taste was recorded in 8% of patients in a study by CDC [8]. Similar to our study they reported a loss of taste and smell in 5.6% and 5.1% of patients respectively while our study showed 2.9% of the patients with anosmia and 1.4% had dysgeusia. Most of our patients had severe COVID-19 disease with the majority needing MICU care and probable physicians’ unawareness about these symptoms could potentially limit accurate data collection for anosmia and dysgeusia.

Our study showed 28% of patients had myalgia at the onset of their illness. Myalgia has been reported in 36% of the patients in which the majority were managed as outpatients due to mild to moderate disease [8]. Skeletal muscle injury has been reported in 10.7% hospitalized COVID-19 patients [9] while our study showed 39.4% of the patients had evidence of skeletal muscle injury (high serum creatine kinase >300U/L) and 15.7% of the patients had evidence of rhabdomyolysis (high serum creatine kinase >1000 U/L).

The skeletal muscle injury rate in our study is far higher than previously reported. Perhaps the more likely explanations are a higher number of patients with underlying comorbidities, especially obesity and chronic kidney disease, longer MICU care, and stay with higher rates of invasive and non-invasive ventilation due to underlying critical COVID-19 disease, multiorgan involvement, and on top of that, all our patients had CK level checked as a part of the initial protocol.

In addition to respiratory and neurological systems, other multi-organ system involvement in COVID-19 patients were kidney injury, liver injury, myocardial injury, heart failure, acute pancreatitis, and uncontrolled blood glucose in our study. The rates of such multi-organ system involvement were higher in our study compared to other studies [9, 23] but similar to another study with critically ill patients [5]. The likely explanations are more sick patients, with a significant number admitted to MICU needing invasive and non-invasive ventilatory support and underlying medical co-morbidities.

This study has several limitations. First, it is a retrospective study that could cause bias in clinical observation. Second, certain patients with milder neurological manifestations as well as lack of physician’s knowledge about COVID-19-related neurological manifestations like loss of taste and smell might not be captured as all data were retrieved from the electronic medical records. Third, several patients had critical diseases with multi-organ failure which limit assessing and eliciting neurological signs in such patients. Fourth, during COVID-19 peak and high admissions rate, neuroimaging like magnetic resonance imaging (MRI) and other diagnostic procedures such as electromyography/nerve conduction velocity were limited due to infection control precautions. In our study, therefore, most of the symptoms were a patient’s subjective descriptions.

## Conclusions

Patients with COVID-19 infection are at higher risk of developing neurological manifestations. The most common COVID-19 related acute neurological manifestations were myalgia, headache, dizziness, and acute ischemic stroke. Prompt recognition, early diagnosis and appropriate management of these manifestations could potentially lead to better patient outcomes in COVID-19 patients. We need prospective randomized control trials to understand and capture all neurological manifestations in COVID-19 patients.
